# Knockdown of CDR1as Decreases Differentiation of Goat Skeletal Muscle Satellite Cells via Upregulating miR-27a-3p to Inhibit ANGPT1

**DOI:** 10.3390/genes13040663

**Published:** 2022-04-09

**Authors:** Bismark Kyei, Emmanuel Odame, Li Li, Liu Yang, Siyuan Zhan, Juntao Li, Yuan Chen, Dinghui Dai, Jiaxue Cao, Jiazhong Guo, Tao Zhong, Linjie Wang, Hongping Zhang

**Affiliations:** Farm Animal Genetic Resources Exploration and Innovation Key Laboratory of Sichuan Province, College of Animal Science and Technology, Sichuan Agricultural University, Chengdu 611130, China; kyeibismark6@gmail.com (B.K.); emmanuelodame123@stu.sicau.com (E.O.); lily@sicau.edu.cn (L.L.); yangqism@gmail.com (L.Y.); siyuan_zhan@163.com (S.Z.); lijuntao1995@163.com (J.L.); chyuan201301@163.com (Y.C.); 71317@sicau.edu.cn (D.D.); jiaxuecao@sicau.edu.cn (J.C.); jiazhong.guo@sicau.edu.cn (J.G.); zhongtao@sicau.edu.cn (T.Z.); wanglinjie@sicau.edu.cn (L.W.)

**Keywords:** siCDR1as, miR-27a-3p, ANGPT1, differentiation

## Abstract

Myogenesis is a complex process controlled by several coding and non-coding RNAs (ncRNAs), such as circular RNAs (circRNAs) that are known to function as endogenous microRNAs (miRNAs) sponges. Cerebellar Degeneration-Related protein 1 antisense (CDR1as) is the most spotlighted circRNA that is known as an miR-7 sponge, which has bloomed circRNAs’ research in animal disease and physiology. Here, we screened for miRNAs and mRNA associated with CDR1as and further characterized their regulatory function during muscle differentiation. We found that a total of 43 miRNAs (including miR-107-3p, miR-125b-5p, miR-140-5p, miR-29a-3p, and miR-27a-3p upregulated) and 789 mRNAs (including ANGPT1, E2F2, CCN1, FGFR1, and MEF2C downregulated) were differentially expressed in goat skeletal muscle satellite cells (SMSCs). Further, knockdown of CDR1as and ANGPT1 inhibited SMSCs differentiation. miR-27a-3p was differentially upregulated after the knockdown of CDR1as in SMSCs. Overexpressed miR-27a-3p decreased SMSCs differentiation. Via RNAhybrid and luciferase, miR-27a-3p was identified to regulate ANGPT1. We discovered that miR-27a-3p has an inverse relationship with CDR1as and decreases the expression level of ANGPT1 during SMSCs differentiation. In summary, our study demonstrates that siCDR1as inhibits myoblast differentiation by downregulating ANGPT1 mRNA via miR-27a-3p in SMSCs.

## 1. Introduction

Skeletal muscle is an important muscle that provides structural support and energy storage, and it is related to the quality and quantity of meat production [1]. Moreover, skeletal muscle is mostly derived from paraxial mesodermal somites and successfully undergoes hyperplasia and hypertrophy processes [2,3]. Myogenesis is the process of skeletal muscle regeneration that starts with the activation of quiescent satellite cells after injury, followed by proliferation, differentiation, and fusion of myoblasts into myotubes [4]. The molecular regulation of the skeletal muscle during embryonic and postnatal development is complex [1]. Besides, it has been widely accepted that several protein-coding genes, such as IGF1R, MEF2C, and ANGPT1, are known to be positively associated with muscle development [5,6,7]. ANGPT1 is known to modulate cell differentiation, survival, and stability [8]. According to Lee et al., ANGPT1 mRNA is known to induce the myogenesis of mouse primary skeletal myoblast [7]. In addition, numerous studies show that ANGPT1 also enhances the survival of cardiac and skeletal myocytes and induces differentiation of satellite cells [8,9]. Muscle development is controlled posttranscriptionally through numerous RNA-binding proteins (RBPs) and also non-coding RNAs (ncRNAs) [10,11,12].

The epigenome is the collection of all of the epigenetic marks on the DNA in a single cell. Besides, it contains different mechanisms, for example, DNA methylation, remodeling, histone tail modifications, microRNAs, and long non-coding RNAs, which work together with several environmental factors, including pathogens, nutrition, and climate, to influence the gene expression profile and the emergence of specific phenotypes [13]. microRNAs (miRNAs) are a small class of non-coding RNAs with a size of about 18–24 nucleotides long that bind to the mRNAs of coding genes to repress their protein production. miRNAs are known to play a crucial role in regulating biological processes, such as myoblast proliferation and differentiation, via controlling targeted mRNAs during muscle development [14]. Among the miRNAs that have been involved in skeletal muscle myogenesis, miR-27a-3p is known to regulate the expression of myostatin and induce myoblast proliferation [15].

In addition to linear transcripts, such as miRNA, lncRNAs, and mRNAs, numerous circular RNAs (circRNAs) responsible for skeletal muscle development have been identified through the introduction of high throughput sequencing technology [16,17,18]. circRNAs are newly discovered non-coding RNA molecules with a closed-loop structure that act as gene regulators in most organisms [19,20]. Moreover, circRNAs also regulate alternative splicing and transcriptional mechanisms, as well as coding micropeptides during several biological processes and diseases [19,21]. Currently, the most attractive and extensively studied function for circRNAs is sponging miRNAs, that is, indirectly elevating miRNA-targeted mRNA genes through a direct sponge of miRNAs and, consequently, relieving the degradation of mRNA caused by the miRNA [19]. Cerebellar Degeneration-Related Protein 1 antisense RNA (CDR1as) is the most well-studied ceRNA as an miR-7 sponge (also known as a sponge for miR-7, ciRS-7). Aside from miR-7, other miRNAs such as miR-135a, miR-876-5p, and miR-1290 have been identified as CDR1as-targeting miRNAs in cancer-related diseases [22,23] and muscle-related diseases [24].

Despite the fact that CDR1as plays an important role in muscle development [5,24], the full transcriptomic effect of CDR1as in myogenic differentiation (including mRNAs and miRNAs), as well as its regulatory mechanisms, is unknown. In this study, we knocked down the expression of CDR1as in goat skeletal muscle satellite cells (SMSCs) and identified differentially expressed patterns of miRNAs and mRNAs associated with the downregulation of CDR1as. Furthermore, we identified that CDR1as controls the expression of ANGPT1 via miR-27a-3p during SMSCs differentiation. These results will help us to fully understand the role of CDR1as in SMSCs development and will provide a paradigmatic example in a systematic study of circRNAs.

## 2. Materials and Methods

### 2.1. Cell Culture

The SMSCs were successfully isolated from the *Longissimus dorsi* (LD) muscles of a newborn goat (3-day old female Nanjiang Brown goat) in our laboratory, as described previously [25]. The SMSCs were cultured in high-glucose Dulbecco’s modified Eagle’s Medium (DMEM) supplemented with 10% FBS (Gibco, Grand Island, NE, USA) and 2% Penicillin & Streptomycin (Invitrogen, Carlsbad, CA, USA) solution (Growth medium, GM) with 5% CO_2_ at 37 °C. Confluent cells were digested with 0.25% trypsin, including 10 mM EDTA, re-suspended in the corresponding medium, and seeded in 6-well plates with a suitable density. To induce differentiation, GM was replaced by DMEM containing 2% HS and 1% Penicillin & Streptomycin (Differentiation medium, DM) when SMSCs amounted to 80–90% confluence. The medium was replaced every 48 h.

### 2.2. Vector Construction and Cell Transfection

The fragment of the ANGPT1 3′ UTR, including the binding sites of miR-27a-3p, was amplified using a forward primer (5′-CCGCTCGAGTACAGTTATCAGCCACCAAG-3′) and a reverse primer (5′-AAATATGCGGCCGCAATAAATACGGGGCCGCAT-3′) and inserted into a psi-CHECK-2 vector (Clontech, Mountain View, CA, USA) at the 3′ end of the Renilla gene using restriction enzymes *Not I* and *Xhol I* (Takara, Dalian, China) and T4 DNA ligase (psi-ANGPT1-3′UTR-WT). The mutant (psi-ANGPT1-3′UTR-Mutant) was generated by mutating complementary to the seed region of the miR-27a-3p using a mutagenic primer. Similarly, the vectors of psi-CHECK-2-CDR1as (pCK-CDR1as-WT/Mutant) were obtained using the same method with a forward primer (5′-CCGCTCGAGCATGTCTTCCAACAATTTC-3′) and a reverse primer (5′-AAATATGCGGCCGCTTTCAGGAAGACCCGAATTG-3′).

Transfection was conducted when SMSCs reached nearly 90% confluence. SMSCs were transfected with siCDR1as versus siNC (50 nM), miR-27a-3p mimic versus NC (50 nM), siANGPT1 versus siNC (50 nM), and ANGPT1 versus psi-CHECK-2 using Lipofectamine 3000 (Invitrogen, Carlsbad, CA, USA) according to the manufacturer’s protocol. The transfection medium was replaced with a growth or differentiation medium 4–6 h post-transfection. The transfected cells were harvested at 48 h (for RNA assay), 72 h (for protein), and immunofluorescence stained at 48 h post-transfection. The chi-miR-27a-3p mimic (TTCACAGTGGCTAAGTTCCG), chi-miR-27a-3p mimic control, siRNA targeting CDR1as (siCDR1as: GTCTACGATATCCAGGGTT), siCDR1as control, siANGPT1, and siANGPT1 control were purchased from Ribobio (RiboBio, Guangzhou, China).

### 2.3. RNA Extraction

Total RNAs were extracted from cells using RNAiso Plus reagent (TaKaRa Bio, Inc., Kusatsu, Japan) and purified using a QIAGEN RNeasy Mini Kit (QIAGEN, Chatsworth, CA, USA) according to the manufacturer’s instructions. RNA degradation and contamination were monitored on 1.5% agarose gels. The purity and concentration of the RNAs were measured using a NanoPhotometer^®^ spectrophotometer (IMPLEN, Los Angeles, CA, USA) and a Qubit RNA Assay Kit in a Qubit^®^ 2.0 Fluorometer (Life Technologies, Carlsbad, CA, USA), respectively. All RNA samples were stored at −80 °C until further use.

### 2.4. miRNA Sequence

After verifying the concentration and purity, the integrity was assessed using an RNA Nano 6000 Assay Kit in a Bioanalyzer 2100 system (Agilent Technologies, Santa Clara, CA, USA). Only samples that had RNA Integrity Number (RIN) scores of >7.5 were used for RNA sequencing. Briefly, small RNAs were reversely transcribed and amplified by PCR. The PCR products were then purified by denaturing polyacrylamide gel electrophoresis (PAGE). A total of 3µg RNA per sample was used as input material for miRNA sampling preparation. miRNA libraries were constructed, sequencing was performed on an Illumina HiSeq 2500 platform (Illumina, San Diego, CA, USA), and 125-bp paired-end reads were generated. miRDeep2 software was used to predict the novel miRNAs with trimmed reads. Then, the reads were aligned to merged pre-miRNA databases (known pre-miRNA from miRBase v21 plus the newly predicted pre-miRNAs) using Novoalign software (v2.07.11) with, at most, one mismatch. We used the most abundant isomiR, the mature miRNA annotated in miRBase, and all isoforms of miRNA (5p or 3p) to calculate miRNA expression. Fold change and p-value were used to calculate the differentially expressed miRNA profiles between the two groups. Hierarchical clustering was performed to generate an overview of the characteristics of expression profiles based on values of significant differentially expressed transcripts.

### 2.5. mRNA Sequencing and Data Processing

Clean reads were obtained by removing reads containing adapters, reads containing over 10% of poly (N), and low-quality reads (>50% of the bases had Phred quality scores ≤ 10) from the raw data. All downstream and upstream analyses were based on clean, high-quality data. The goat reference genome and gene model annotation files were downloaded from the NCBI database (CHIR_1.0, NCBI) [26]. The index of the reference genome was built using Bowtie v2.0.6 [27,28], and paired-end clean reads were aligned to the reference genome using TopHat v2.0.14 [29]. The mapped reads from each library were assembled with Cufflinks v2.2.1 [30]. We used the reference annotation-based transcript (RABT) assembly method in cufflinks v2.2.1 to construct and identify mRNA transcripts from the TopHat2 alignment results. Hierarchical clustering was performed to generate an overview of the characteristics of expression profiles based on values of significant differentially expressed transcripts.

### 2.6. KEGG Pathway Analysis

Kyoto Encyclopedia of Genes and Genomes (KEGG) analysis of DEGs was performed with KOBAS software [31] using a hypergeometric test. KEGG pathways with a Q value < 0.05 were considered significantly enriched.

### 2.7. Validation of RNA-Seq Data by qRT-PCR

Total RNAs were extracted from SMSCs using RNAiso Plus reagent (TaKaRa Bio, Inc., Kusatsu, Japan). For quantitative Real-Time PCR (qRT-PCR) of mRNA, all PCR primers were designed at or just outside exon/exon junctions to avoid the amplification of residual genomic DNA using Primer-BLAST on the NCBI website, and specificity was determined using BLASTN. A total of 1µg of total RNA was reversely transcribed into cDNA by using the PrimeScriptTM RT Reagent Kit with gDNA Eraser (TaKaRa, Otsu, Japan). Using these cDNAs as templates, expression levels of genes were quantified by qRT-PCR in a Bio-Rad CFX96 system (Bio-Rad, Hercules, CA, USA) with SYBR Premix Ex TaqTM II (TaKaRa, Otsu, Japan) according to the manufacturer’s protocols. Three samples were collected for each treatment, and each sample was at least triplicated. The PCR protocol was as follows: denaturation at 95 °C for 30 s, followed by 40 cycles of 95 °C for 20 s, 60 °C for 20 s, and 72 °C for 30 s. The 2^−^^ΔΔCt^ procedure was used to calculate the relative expression levels of mRNAs, with GAPDH as an internal control [32]. For qRT-PCR of miRNA, reverse transcription was done using the First-Strand cDNA Synthesize (TaKaRa, Mount View, CA, USA). For real-time PCR, all reactions were performed in triplicate with SYBR Premix Ex TaqTM II (TaKaRa, Otsu, Japan) under the following conditions: 10 s at 95 °C for initial denaturation, followed by 39 cycles of 95 °C for 5 s and 60 °C for 20 s, followed by a melting curve of 65 °C to 95 °C for 5 s. The expression levels of U6 were used to normalize the expression levels of the gene of interest. Primers for the mRNAs and miRNAs were designed using Primer-BLAST (http://www.ncbi.nlm.nih.gov/tools/primers-blast, accessed on 15 February 2020) (Supplementary Table S1A,B).

### 2.8. Western Blot Assay

To measure the protein level of MyoD, the total proteins from cultured SMSCs were extracted using the Total Protein Extraction Kit (BestBio, Shanghai, China) and quantified by employing BCA Protein Quantitation Kit (BestBio, Shanghai, China) according to the manufacturer’s instructions. In brief, with ~20 μg of protein per sample, Western blotting was performed by separating protein on a 12% SAS-PAGE, transferring to a PVDF (Millipore, Burlington, MA, USA), blocking with 5% milk in TBS, and then incubating the membrane sequentially with the primary anti-mouse MyoD (1:1000) (Abcam, Bristol, UK) or MyHC (1:1000) (Minneapolis, MN, USA) and the secondary antibody IgG (Beyotime, Shanghai, China). Eventually, we measured the enhanced chemiluminescence signal (ECL) (Solarbio, Beijing, China) after adding horseradish peroxidase (HRP) (Bio-Rad, CA, USA) and the GAPDH antibody (1:1000, mouse) (Boster, Wuhan, China) as a loading control.

### 2.9. EdU Proliferation Assay

When cells reached approximately 70% confluence, the SMCSs were transfected as described above. Then, the cells were cultured in GM for 24 h post-transfection and then incubated for 2 h in a medium containing 50 μM EdU (RiboBio, Guangzhou, China) before immunostaining. Next, the cells were fixed, permeabilized, and stained following the manufacturer’s instructions. A minimum of three images per group was obtained using a fluorescence microscope (Olympus, Tokyo, Japan). The percentage of EdU-positive cells was calculated by dividing the number of nuclei incorporating EdU by the number of total nuclei.

### 2.10. Immunofluorescence Analyses

SMSCs (~2 × 10^4^ cells per well) were seeded in 3.5-cm petri dishes and cultured in DM. The SMSCs were tri-washed with ice precooling PBS after removing the culture medium, fixed with 4% paraformaldehyde for 15min at room temperature, washed 3 times again with 1mL PBS after getting rid of paraformaldehyde, permeabilized with 1mL 0.5% Triton X-100 at 4 °C for 10 min, washed with PBS (3 times), and blocked with 1 mL 2% bovine serum albumin (BSA) at 37 °C for 30 min. Then, the cells were incubated with anti-mouse MyHC (1: 200, Fa. R&D Systems, Minneapolis, MN, USA) overnight at 4 °C, washed 3 times with PBS (5 min each), and subsequently incubated with secondary antibodies Cy3_IgG (H+L) (1: 200, Solarbio, Shanghai, China) at 37 °C for 2 h, and triple washed with PBS again. Finally, cells were stained with 0.05 μg/mL DAPI (4′,6′-diamidino-2-phenylindole; Invitrogen) at 37 °C for 10 min in a humidified dark chamber. After being washed 3 times with 1mL PBS, the images were observed under a fluorescent microscope (Olympus, Japan). The ImageJ software (National Institutes of Health) was employed to count cells in each photo, including the total number of nuclei (on the DAPI channel), the number of nuclei surrounded by the MyHC signal, and the number of MyHC+ myotubes. Then, the percentage of MyHC positive cells was calculated as the ratio of the number of nuclei surrounded by the MyHC signal to the total number of nuclei. For each treatment, at least three samples were independently performed, and five areas per sample were randomly selected.

### 2.11. Luciferase Assay Activity

To determine the relationship between ANGPT1 and miR-27a-3p, RNAhybrid was used to predict the binding sites between ANGPT1 and miR-27a-3p. A partial sequence of ANGPT1 3′ UTR (harboring the binding site for miR-27a-3p) was amplified and cloned into a psiCHECK-2 vector (Clontech, Mountain View, CA, USA), downstream renilla luciferase gene (Rluc). Mutant derivatives (ANGPT1 3′ UTR Mutant) were obtained by mutating miR-27a-3p binding sites and were generated by Sangon Biotech (Shanghai, China). Similarly, the vectors of psi-CHECK-2-CDR1as (pCK-CDR1as-WT/Mutant) were obtained using the same method. The transfection efficiency was evaluated according to the firefly luciferase gene (Fluc) seeded on the same plasmid. The luciferase reporter assays were co-transfected into SMSCs using Lipofectamine 2000. The activities of both luciferases were measured 72 h after transfection using the Dual-Luciferase Reporter Assay Kit (Promega, Madison, WI, USA).

### 2.12. Statistical Analyses

Data are expressed as mean ± SEM. All statistical analyses were performed using GraphPad Prism 6.01. Unpaired students’ *t*-tests were used for comparisons between two groups. *p* < 0.05 was considered a statistically significant difference among the means.

## 3. Results

### 3.1. Overview of mRNA and miRNA Sequencing Data Associated with CDR1as

To predict miRNAs and mRNAs associated with CDR1as in SMSCs, we in vitro cultured SMSCs isolated from the Longissimus dorsi (LD) muscles of a newborn Nanjiang Brown goat and knocked down the expression of CDR1as (siCDR1as 50 nM) with three biological replicates for each treatment. Cells were harvested at 48 h after transfection, and the total RNA was extracted to construct the cDNA libraries individually for miRNA-seq and mRNA-seq using an Illumina HiSeq 2500 platform and 125 bp paired-end reads.

After removing low-quality sequences and adapters, an average of 15,350,087 and 14,614,375 miRNAs were produced from raw and clean reads, respectively (Table 1). In addition, an average of 59,821,102 raw reads and 58,552,463 clean reads of mRNAs were generated (Table 2).

To explore the relationship of genes between samples, Pearson’s correlation coefficient (PCC) of mRNAs and miRNAs expression levels of siCDR1as-1, 2, and 3 in SMSCs were calculated and used to generate a correlation chart. As shown, the correlation coefficient of the siCDR1as-1, 2, and 3 in SMSCs ranged from 0.96 to 0.99 (an average of 0.98), indicating that the samples replicated very well biologically (Supplementary Figure S1A,B).

### 3.2. Differentially Expressed (DE) miRNAs in SMSCs Transfected with siCDR1as

**Figure 1:**
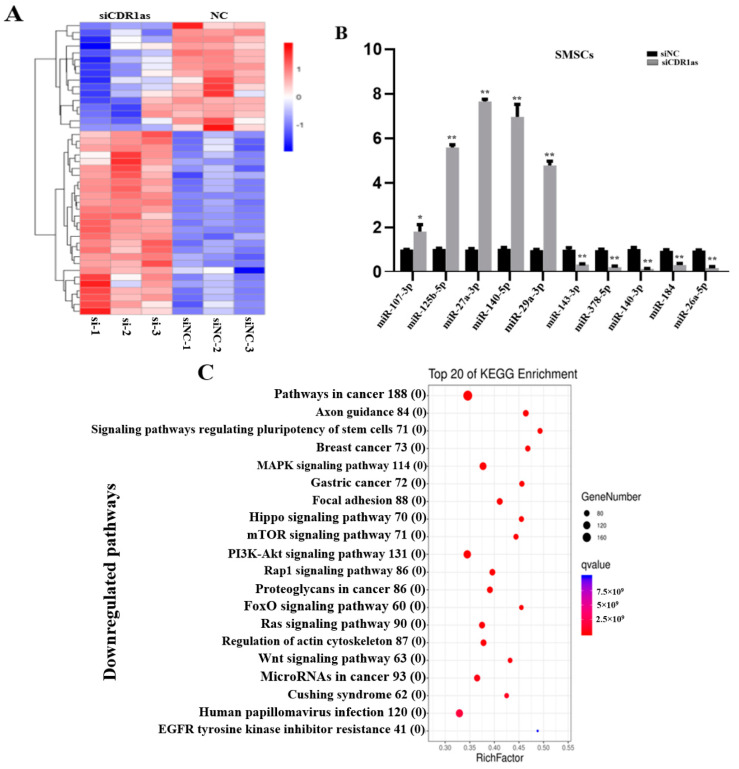
Differentially expressed miRNAs in siCDR1as and siNC (SMSCs). (**A**) Sequencing analysis for miRNAs was performed from siCDR1as (*n* = 3) and siNC (*n* = 3) of SMSCs. Hierarchical cluster analysis of DE miRNAs: bright red, overexpression; white, no change; bright blue, under-expression. (**B**) Differential expression of 10 representative miRNAs was validated in siCDR1as and siNC of SMSCs by qRT-PCR (*n* = 10 per group). The number of ‘’*’’ indicates the level of significance (**, *p* < 0.01, *, *p* < 0.05). (**C**) KEGG analysis of the upregulated miRNA-mRNA network. The rich factor indicates the ratio of the number of DEGs mapped to a certain pathway to the total number of genes mapped to this pathway.

miRNA sequencing was performed to identify the miRNA expression profiles of SMSCs transfected with siCDR1as. According to the database, a total of 529 miRNAs were detected in SMSCs samples, among which 43 miRNAs, including 16 downregulated (15 known and 1 novel) and 27 upregulated miRNAs (24 known miRNAs and 3 novels), were identified (padj < 0.05) (Figure 1A; Supplementary Table S2). In addition, the expressions of five upregulated (miR-107-3p, miR-125b-5p, miR-140-5p, miR-29a-3p, and miR-27a-3p) and five downregulated (miR-143-3p, miR-378-5p, miR-140-3p, miR-184, and miR-26a-5p) miRNAs were randomly selected and validated by qRT-PCR to confirm the RNA-sequenced data (Figure 1B).

Moreover, from the data, a total of 1,234 mRNAs were known to be targeted by the siCDR1as-upregulated miRNAs, whilst 912 mRNAs were known to be targeted by the downregulated miRNAs. To further study the putative functions of DE miRNAs, all the potential target mRNAs were then filtered, and the miRNA-target expression correlation of upregulated or downregulated miRNAs was used to perform KEGG pathway analysis separately (Q value < 0.05). MAPK signaling pathway, Focal adhesion, PI3K-AKT signaling pathway, Rap1 signaling pathway, Wnt signaling pathway, and Hippo signaling pathways, which are associated with muscle development, were among the top 20 most enriched KEGG pathways of the upregulated-miRNA targeted genes (Figure 1C). However, the PI3K-AKT signaling pathway, FoxO signaling, and MAPK signaling pathways were among the top enrichments of mRNAs targeted by downregulated miRNAs, too (Supplementary Figure S2). These results show that the upregulated miRNAs associated with the knockdown of CDR1as may be related to muscle development and growth.

### 3.3. DE mRNAs in SMSCs Transfected with siCDR1as

**Figure 2:**
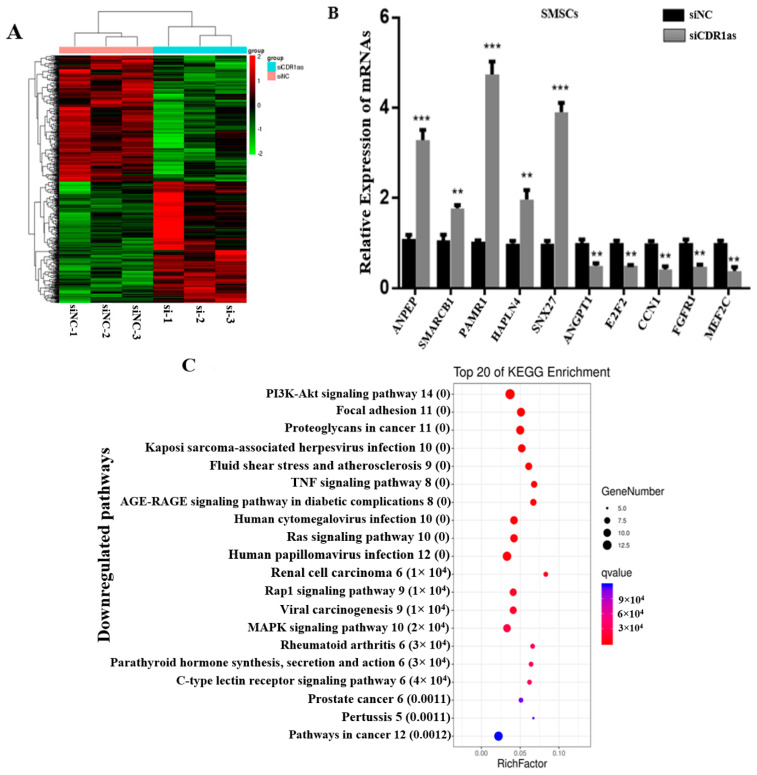
Expression profile of mRNAs in siCDR1as and siNC (SMSCs). (**A**) Sequencing analysis for mRNAs was performed with RNA extracted from siCDR1as (*n* = 3) and siNC (*n* = 3) SMSCs. Hierarchical cluster analysis of significantly expressed mRNAs: bright green, under-expression; bright red, overexpression. (**B**) Ten differentially expressed representative mRNAs were validated in SMSCs transfected with siCDR1as and siNC by qRT-PCR (*n* = 10 per group). GAPDH was used as an internal control. The number of ‘’*’’ indicates the level of significance (***, *p* < 0.001, **, *p* < 0.01). (**C**) KEGG of the downregulated mRNAs with the top 20 enrichments. The bubble color and size correspond to the Q value and gene number enriched in the pathway. The rich factor indicates the ratio of the number of DEGs mapped to a certain pathway to the total number of genes mapped to this pathway.

From mRNA expression profiling data, a total of 789 mRNAs (Figure 2A) were differentially expressed in SMSCs transfected with siCDR1as (*p* < 0.05), of which 401 mRNAs were downregulated (316 known protein-coding genes and 85 novel transcripts) and 388 mRNAs were upregulated (277 protein-coding genes, 112 novel transcripts). Subsequently, qRT-PCR was used to confirm five randomly selected upregulated and downregulated DE mRNAs after the knockdown of CDR1as (Figure 2B; Supplementary Table S3). Some myogenic genes, including ANGPT1 [33], E2F2 [34], CCN1 [35], fibroblast growth factor receptor 1 (FGFR1) [36], and MEF2C [37], were downregulated according to the sequencing data.

Further, KEGG pathway analysis was performed based on the DE mRNA genes (Q value < 0.05). The result shows that among the top 20 most enriched pathways of the downregulated genes in the siCDR1as samples, some well-known muscle-related pathways, including the PI3K-AKT signaling pathway, Focal adhesion, Rap1 signaling pathway, and MAPK signaling pathway, were identified (Figure 2C). Besides, ANGPT1, MEF2C, and FGFR1 are among the genes responsible for the PI3K-AKT signaling pathway, the Rap1 signaling pathway, and the MAPK signaling pathway. Moreover, the genes upregulated by siCDR1as were mainly enriched in fatty acid biogenesis and metabolism (Supplementary Figure S3). These results indicate that the knockdown of CDR1as is related to the downregulation of myogenic genes.

### 3.4. Effect of miR-27a-3p on SMSCs

**Figure 3:**
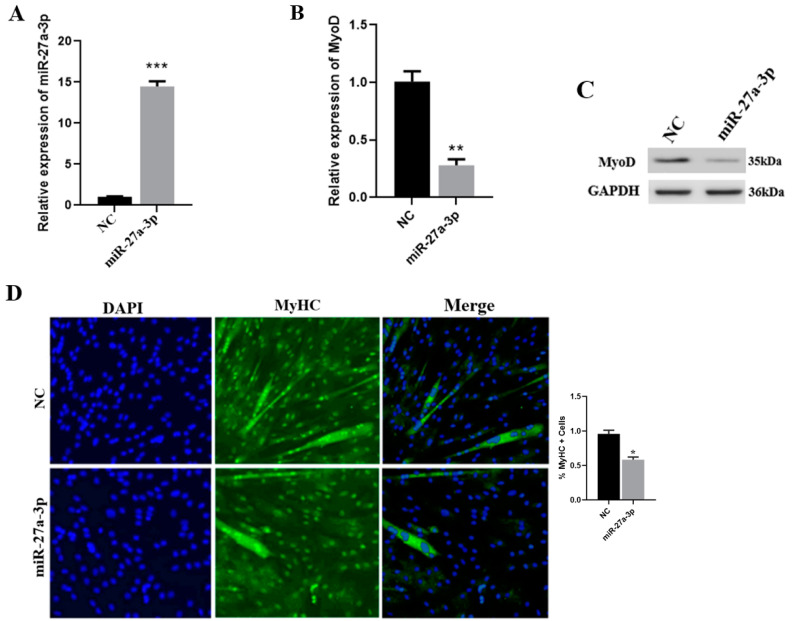

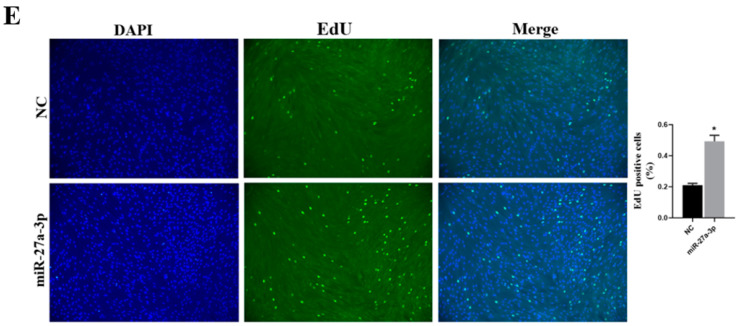
Overexpression of miR-27a-3p increases proliferation and reduces SMSCs differentiation. (**A**) qRT-PCR analysis to determine the expression of miR-27a-3p in SMSCs. (**B**) qRT-PCR analysis to determine the expression level of MyoD in SMSCs after transfecting with miR-27a-3p mimic. (**C**) The protein level of MyoD was analyzed by Western blotting after overexpression of miR-27a-3p mimic in SMSCs. (**D**) Representative immunofluorescence images of SMSCs after transfected with miR-27a-3p mimic and (or) NC and maintained in differentiating for 48 h. (**E**) Cell proliferation was detected with 5-ethynyl-20-deoxyuridine (EdU) and the proliferation rate was represented by the percentage of EdU positive cells. The number of ‘’*’’ indicates the level of significance (***, *p* < 0.001, **, *p* < 0.01, *, *p* < 0.05). The *p* values were analyzed by Student’s *t*-test; *p* < 0.05.

miR-27a-3p was identified as a differentially upregulated miRNA after the knockdown of CDR1as in SMSCs. To explore the function of miR-27a-3p on SMSCs differentiation and its relationship with CDR1as, we first examined the expression level of miR-27a-3p in SMSCs transfected with miR-27a-3p mimic by qRT-PCR (Figure 3A). The RNA expression level of MyoD was significantly decreased in SMSCs transfected with miR-27a-3p mimic (Figure 3B). Besides, Western blotting analysis shows that miR-27a-3p reduced the expression level of MyoD as compared to the NC in SMSCs (Figure 3C). We detected the effect of miR-27a-3p on myogenic differentiation via immunofluorescence assay and found that miR-27a-3p inhibited myotube formation (Figure 3D). In addition, the EdU proliferation assay shows that miR-27a-3p enhances the proliferation of SMSCs (Figure 3E). These results indicate that miR-27a-3p increases proliferation and decreases the differentiation of SMSCs.

### 3.5. The Functional Role of ANGPT1 in SMSCs and Its Relationship with miR-27a-3p

**Figure 4:**
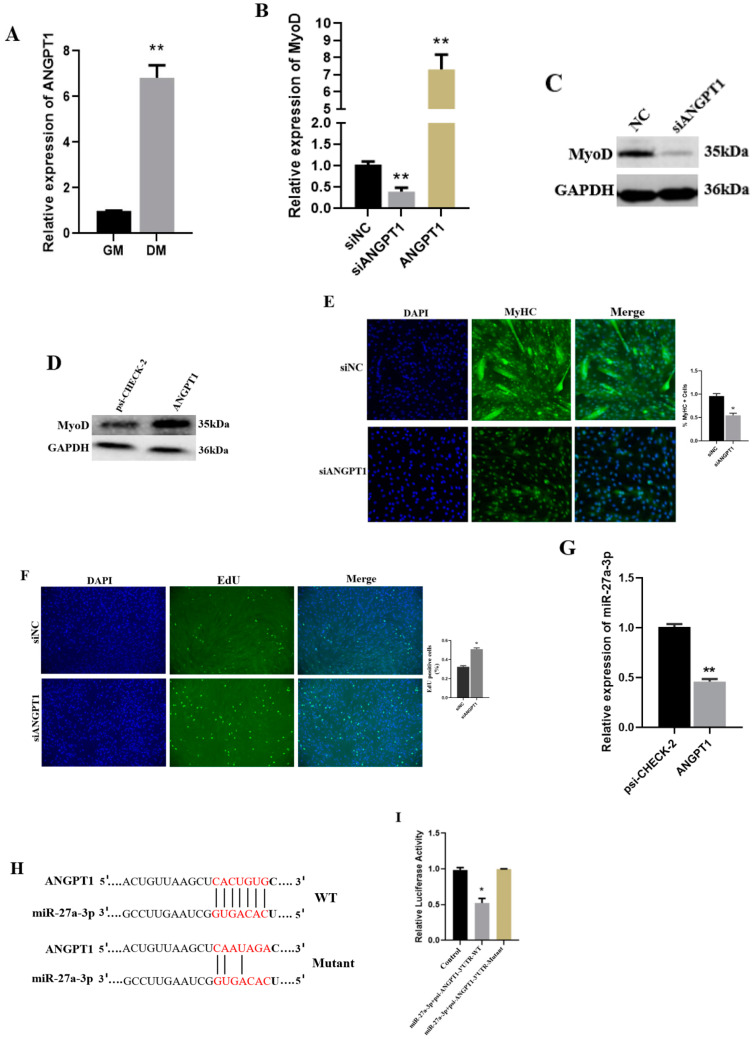
The function of ANGPT1 and its regulatory role in SMSCs. (**A**) qRT-PCR analysis to determine the expression level of ANGTP1 in the GM and DM of SMSCs. (**B**) qRT-PCR analysis of MyoD expression after knockdown and overexpression of ANGPT1. (**C**,**D**) The protein level of MyoD was analyzed by Western blotting after knockdown and overexpression of ANGPT1 in SMSCs. (**E**) Representative immunofluorescence images of SMSCs after transfected with siANGPT1 and (or) siNC and maintained in differentiating for 48 h. (**F**) Cell proliferation was detected with 5-ethynyl-20-deoxyuridine (EdU), and the proliferation rate was represented by the percentage of EdU positive cells. (**G**) qRT-PCR analysis to determine the relationship between miR-27a-3p and ANGPT1 in differentiated SMSCs. (**H**) The binding sites on ANGPT1 3′ UTR. (**I**) The luciferase activity of SMSCs transfected with miR-27a-3p mimic and psi-ANGPT1-3′UTR-WT/Mutant. The Renilla luciferase activity was normalized to the firefly luciferase activity. The number of ‘’*’’ indicates the level of significance (**, *p* < 0.01, *, *p* < 0.05). The *p* values were analyzed by Student’s *t*-test; *p* < 0.05.

To determine the influence of CDR1as and/or miR-27a-3p on ANGPT1 during myoblast differentiation, we first determined the mRNA level of ANGPT1 in GM and DM (Figure 4A). Knockdown of ANGTP1 decreased the RNA expression level of MyoD while overexpressing ANGTP1 upregulated the RNA expression levels of MyoD (Figure 4B). Moreover, Western blotting analysis indicates that the protein level of MyoD was decreased and enhanced after the knockdown (Figure 4C) and overexpression (Figure 4D) of ANGPT1, respectively. An immunofluorescence assay shows that inhibition of ANGPT1 reduced SMSC’s myotube formation (Figure 4E). To determine the effect of ANGPT1 on cell proliferation, we pretreated SMSCs with siANGPT1 and siNC using EdU. We found that siANGPT1 increases the number of Edu-positive cells during SMSC proliferation (Figure 4F). miR-27a-3p negatively correlates with ANGPT1 relative RNA expression levels in differentiating SMSCs (Figure 4G). RNAhybrid was used to predict the binding sites between miR-27a-3p and ANGPT1 (Figure 4H). Through a luciferase assay, we found that miR-27a-3p significantly reduced the relative luciferase activity in SMSCs co-transfected with miR-27a-3p mimic and psi-ANGPT1-3′ UTR WT as compared to the psi-ANGPT1-3′ UTR-Mutant (Figure 4I). These confirm that ANGPT1 is associated with muscle differentiation and can be regulated by miR-27a-3p.

### 3.6. CDR1as Knockdown Inhibits Differentiation of SMSCs

**Figure 5:**
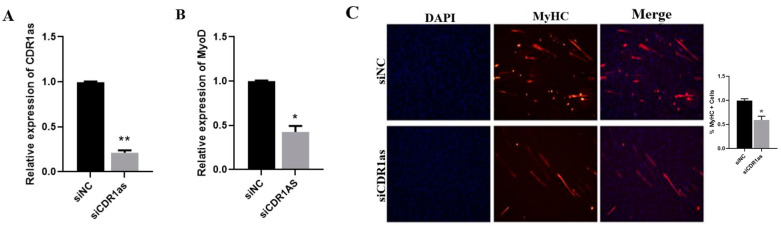

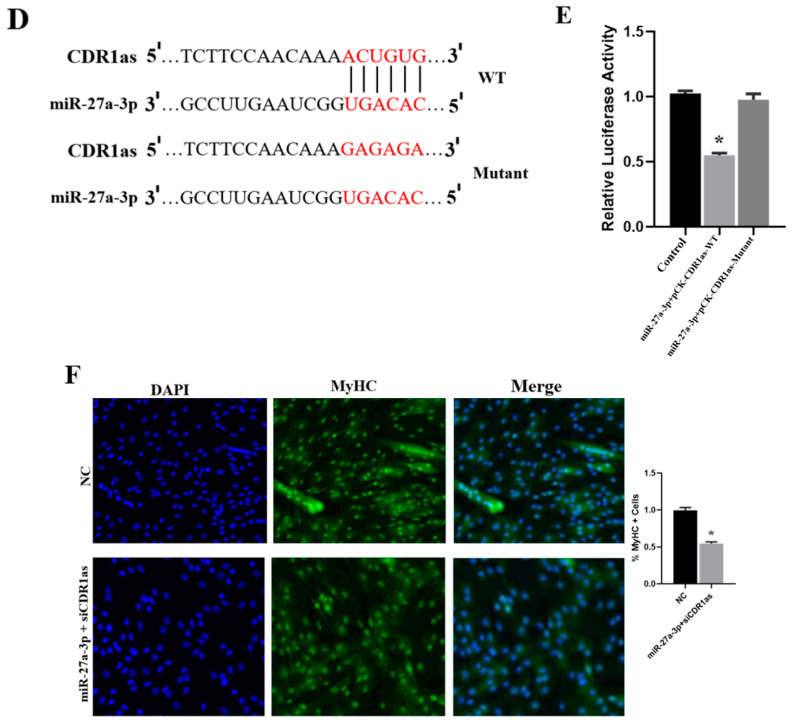
siCDR1as inhibits ANGPT1 via upregulation of miR-27a-3p. (**A**) Validation of the RNA expression levels of CDR1as in SMSCs transfected with siCDR1as. (**B**) RNA expression levels of MyoD after transfecting with siCDR1as. (**C**) MyHC immunofluorescence was decreased during siCDR1as. (**D**) The binding site analysis between CDR1as and miR-27a-3p. (**E**) Relative Luciferase activity of putative miR-27a-3p to pCK-CDR1as-WT/Mutant. (**F**) SMSCs transfected with siCDR1as+miR-27a-3p, cell differentiation was detected by immunofluorescence (MyHC) and MyHC positive cells. The number of ‘’*’’ indicates the level of significance (**, *p* < 0.01, *, *p* < 0.05). The *p* values were analyzed by Student’s *t*-test; *p* < 0.05.

According to our previous results, CDR1as plays a critical role in myogenesis by functioning as a molecular sponge for miR-7 [5]. To identify whether CDR1as is associated with myoblast differentiation, we systematically cultured SMSCs and knocked down the expression of CDR1as (Figure 5A) by using siCDR1as. Furthermore, deficiency of CDR1as inhibited the expression of MyoD (Figure 5B) and myoblast differentiation (Figure 5C). RNAhybrid and Targetscan were used to predict the miR-27a-3p recognition sequence on goat CDR1as (Figure 5D). The luciferase assay revealed that miR-27a-3p significantly inhibited Rluc expression of pCK-CDR1as-WT (Figure 5E). Using immunofluorescence assays, we found that the simultaneous knockdown of CDR1as and the overexpression of miR-27a-3p decreases myogenic differentiation (Figure 5F). Conclusively, we propose that siCDR1as decreases SMSCs differentiation via upregulating miR-27a-3p to inhibit ANGPT1. These results indicate CDR1as has a significant role to play during myogenesis.

## 4. Discussion

Recent studies indicate that muscle development is associated with several coding and non-coding genes, including circRNAs, miRNAs, and mRNAs [19]. For instance, circFGFR4 induces myoblast differentiation through the upregulation of Wnt3a via the downregulation of miR-107 [38]. Moreover, *circSVIL* inhibits miR-203 by increasing the expression level of MEF2C during muscle differentiation [37]. Therefore, studying the transcriptomic view of CDR1as, its regulatory mechanism associated with miR-27a-3p, and ANGPT1 during muscle differentiation in SMSCs, may contribute to the understanding of genes involved in myogenesis. In this study, we found that upregulation of miR-27a-3p by siCDR1as decreases SMSCs differentiation through the downregulation of ANGPT1.

CDR1as functions in tumors and cancer have been extensively studied [39,40,41]. We previously confirmed that CDR1as promotes the myogenic differentiation of SMSCs [5]. However, there is limited knowledge about CDR1as in muscle development. Besides, the number of miRNAs and mRNAs associated with CDR1as in SMSCs remains unknown. In our studies, we knocked down the expression of CDR1as in SMSCs to determine the differentially expressed downregulated and upregulated miRNAs and mRNAs. A total of 43 miRNAs were differentially expressed, consisting of 27 upregulated and 16 downregulated after the knockdown of CDR1as in SMSCs. Furthermore, 789 mRNAs were differentially expressed, comprising 401 downregulated mRNAs and 388 upregulated mRNAs. Some significantly downregulated mRNAs, such as MEF2C [42], ANGPT1 [33], E2F2 [34], CCN1 [35], and FGFR1 [36], are shown to regulate muscle development.

Skeletal muscle development is a gradual process that involves proliferation, differentiation, and fusion, and it is controlled by several signaling pathways. Moreover, myogenesis is controlled by various signaling regulatory networks, including the PI3K-AKT signaling pathway, Focal adhesion, Rap1 signaling pathway, MAPK signaling pathway, and FoxO signaling [43,44,45,46]. We performed a KEGG pathway analysis to study the putative functions of DEGs. According to the KEGG analyses, the most enriched pathways associated with the downregulated mRNAs were the (PI3K)-AKT signaling pathway, Focal adhesion, Rap1 signaling pathway, and MAPK signaling pathway. Furthermore, miR-106a-5p has been shown to inhibit the myogenic differentiation of C2C12 myoblasts by downregulating the PI3K/AKT signaling pathway [43]. According to Jiang et al., the knockdown of PI3-Kinase or its downstream target AKT prevents muscle differentiation in cell culture, while activation of PI3-Kinase and Akt induces myogenic differentiation [47,48]. According to a study conducted, Focal adhesion, along with ECM, serves as the signaling hub for several intracellular pathways related to cell proliferation and differentiation [44]. Furthermore, Focal adhesion plays a significant regulatory role in biological processes, including muscle differentiation and striated muscle tissue development [46]. Rap 1 is a small GTPase that regulates many processes, including cell–cell junction tightening [49], cell polarity, and cell adhesion [50]. In addition, Rap 1 signaling is known to be related to the β-adrenergic signaling pathway, which plays a crucial role in skeletal muscle growth and development [51]. The MAPK signaling pathway appears to be involved in the regulation of muscle stem cell regeneration and proliferation [45]. Moreover, the MAPK signaling pathway plays a significant role during myoblast differentiation [52,53]. Aside from the above-mentioned signaling pathways, the KEGG pathways of the upregulated-miRNA target mRNAs also include WNT and Hippo signaling pathways, which are also known to be among the significantly enriched pathways. Besides, the WNT signaling pathway controls satellite cell differentiation and regeneration [54,55]. The Hippo signaling pathway is also known to play an important role in myogenesis [56,57]. Furthermore, the FoxO signaling pathway is an essential pathway related to muscle development at different stages [46].

miRNAs are known to play significant roles in muscle development [5,58]. In this study, we chose miR-27a-3p, whose expression level was higher than other miRNAs in SMSCs during siCDR1as. We explored the role of miR-27a-3p in myogenesis. According to Cui et al., overexpression of miR-27a-3p inhibited, whereas knockdown of miR-27a-3p enhanced the differentiation of lung fibroblasts into myofibroblasts [59]. Furthermore, Zhang et al., stated that overexpression of miR-27a during porcine myoblast differentiation decreases the expression of myogenin, which is a key marker gene in myoblast differentiation [60]. Through examining the effect and the regulatory mechanism of miR-27a-3p in differentiating SMSCs, we found that overexpression of miR-27a-3p significantly decreased the formation of myotubes. In addition, miR-27a-3p significantly reduced the RNA and protein expression of MyoD, hence revealing its function as a negative myogenic differentiation regulatory factor during SMSC differentiation. miR-27a also enhances C2C12 cell proliferation during myogenesis [61,62]. We found that miR-27a-3p increases SMSC proliferation. Significantly, miR-27a-3p plays an important role in muscle development by decreasing myotube formation.

ANGPT1 plays an essential role during myogenesis by enhancing skeletal muscle regeneration [7,33]. According to our results, the EdU proliferation assay revealed that knockdown of ANGPT1 increased the proliferation of SMSCs compared to its control. Besides, knockdown of ANGPT1 is shown to decrease the formation of myotubes, as well as the protein level of MyoD, while overexpression of ANGPT1 significantly enhances the expression of MyoD and myotube formation. ANGPT1 was identified as a target of miR-27a-3p using RNAhybrid, a dual-luciferase reporter assay, and qRT-PCR in SMSCs. The results indicate that myoblast differentiation is permitted by blocking miR-27a-3p proliferation. We hypothesize that the effect of siCDR1as on myoblast differentiation is caused by miR-27a-3p-mediated ANGPT1 regulation. Therefore, we can deduce that siCDR1as decreases ANGPT1 expression by upregulating miR-27a-3p during myogenesis. The regulatory network siCDR1as/miR-27a-3p/ANGPT1 highlights the mechanisms of non-coding RNAs in regulating muscle development.

## 5. Conclusions

In summary, we found that knockdown of CDR1as enhanced the activity of miR-27a-3p and decreased the differentiation of SMSCs. Our results suggest that miR-27a-3p inhibits the activity of ANGPT1 during the differentiation of SMSCs.

## Figures and Tables

**Table 1:** Summary of reads from raw data and clean read for miRNAs (siCDR1as).

Sample	Raw Reads	Clean Reads	Clean Reads (%)
siCDR1as-1	15,249,256	14,876,516	97.56%
siCDR1as-2	15,109,243	14,776,717	97.80%
siCDR1as-3	16,302,005	16,044,248	98.42%
siNC-1	17,583,292	17,101,617	97.26%
siNC-2	15,392,007	15,172,466	98.57%
siNC-3	16,631,937	16,245,764	97.68%

**Table 2:** Summary of reads from raw data and clean read for mRNAs (siCDR1as).

Sample	Raw Reads	Clean Reads	Clean Reads (%)
siCDR1as-1	51,865,796	51,084,670	98.49%
siCDR1as-2	64,928,768	63,448,702	97.72%
siCDR1as-3	62,668,742	61,124,018	97.53%
siNC-1	71,050,762	69,247,162	97.46%
siNC-2	60,328,494	58,720,344	97.33%
siNC-3	73,310,062	70,808,056	96.59%

## Data Availability

The datasets used and/or analyzed during the current study are available from the corresponding author on reasonable request.

## Supplementary Materials

The following supporting information can be downloaded at: https://www.mdpi.com/article/10.3390/genes13040663/s1, Figure S1: (A–B). siCDR1as Pearson correlation chart. The abscissa and the ordinate were the respective samples, and the abscissa and the ordinate of each patch represented the correlation of siCDR1as samples. Importantly, two completely related genomes had a value of 1. The closer the relative value is to 1, the larger the Pearson correlation coefficient (PCC). Conversely, the closer to 0 the relative value was, the smaller the PCC.; Figure S2: Downregulated miRNAs target mRNAs. Bubble color and size correspond to the Q value and gene number enriched in the pathway. The rich factor indicates the ratio of the number of DEGs mapped to a certain pathway to the total number of genes mapped to this pathway. Figure S3: KEGG of the upregulated mRNAs with the top 20 enrichment. Bubble color and size correspond to the Q value and gene number enriched in the pathway. The rich factor indicates the ratio of the number of DEGs mapped to a certain pathway to the total number of genes mapped to this pathway. Table S1: (A). Primers for qPCR, related to experimental procedures qPCR primers for analysis of mRNA (siCDR1as); (B). qPCR primers for analysis of miRNA (siCDR1as). Table S2: 43 miRNAs that are critical for muscle cells after inhibition of CDR1as. Table S3: The deregulated expression of mRNAs in microarray in SMSCs (siCDR1as).
