# Supplementary material for: Knockdown of CDR1as Decreases Differentiation of Goat Skeletal Muscle Satellite Cells via Upregulating miR-27a-3p to Inhibit ANGPT1
Source: Genes (Basel). 2022 Apr 9;13(4):663. doi: 10.3390/genes13040663 (PMC9026999; doi:10.3390/genes13040663)
Supplement: Supplementary file 1 [file genes-13-00663-s001.zip › genes-1652043-supplementary.pdf]

Supplementary information

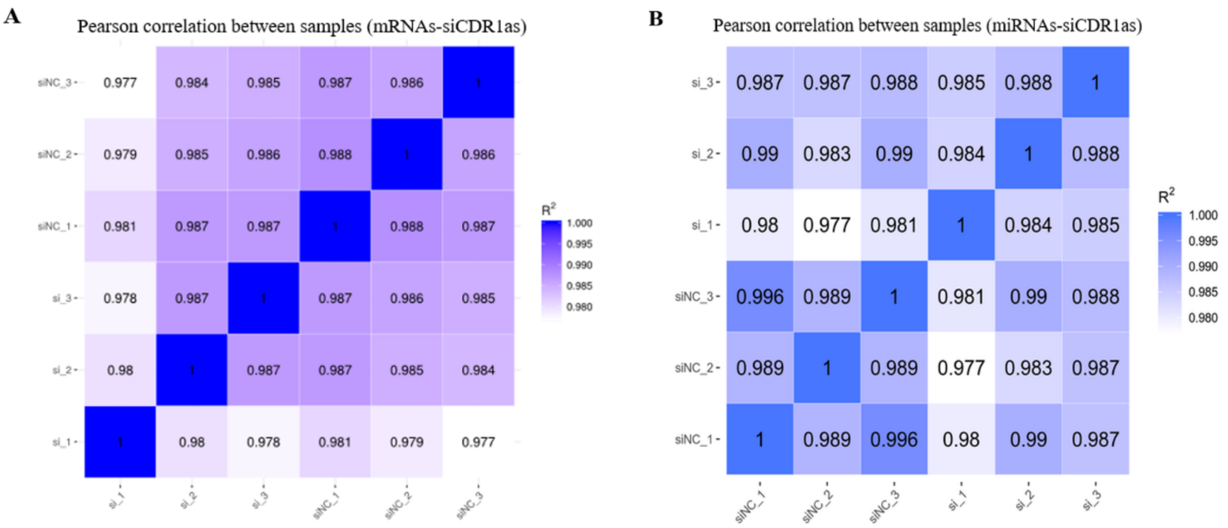

Supplementary Figure S1. (**A**,**B**) siCDR1as Pearson correlation chart. The abscissa and the ordinate were the respective samples, and the abscissa and the ordinate of each patch represented the correlation of siCDR1as samples. Importantly, two completely related genomes had a value of 1. The closer the relative value is to 1, the larger the Pearson correlation coefficient (PCC). Conversely, the closer to 0 the relative value was, the smaller the PCC.

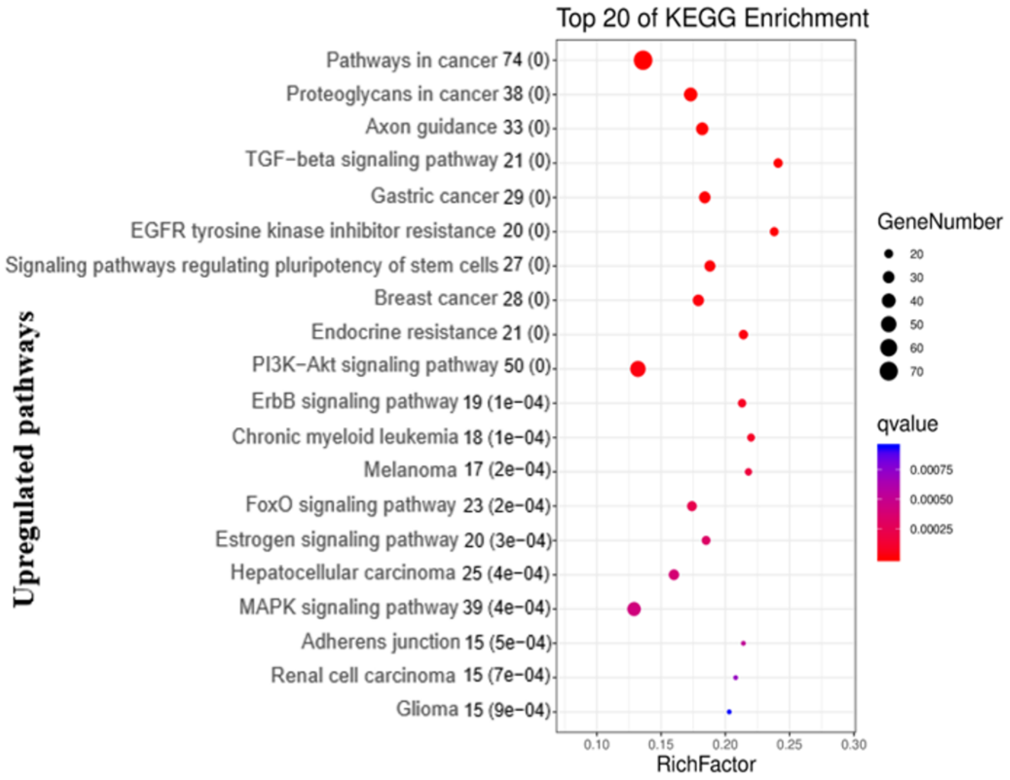

Supplementary Figure S2. Downregulated miRNAs target mRNAs. Bubble color and size correspond to the Q value and gene number enriched in the pathway. The rich factor indicates the ratio of the number of DEGs mapped to a certain pathway to the total number of genes mapped to this pathway.

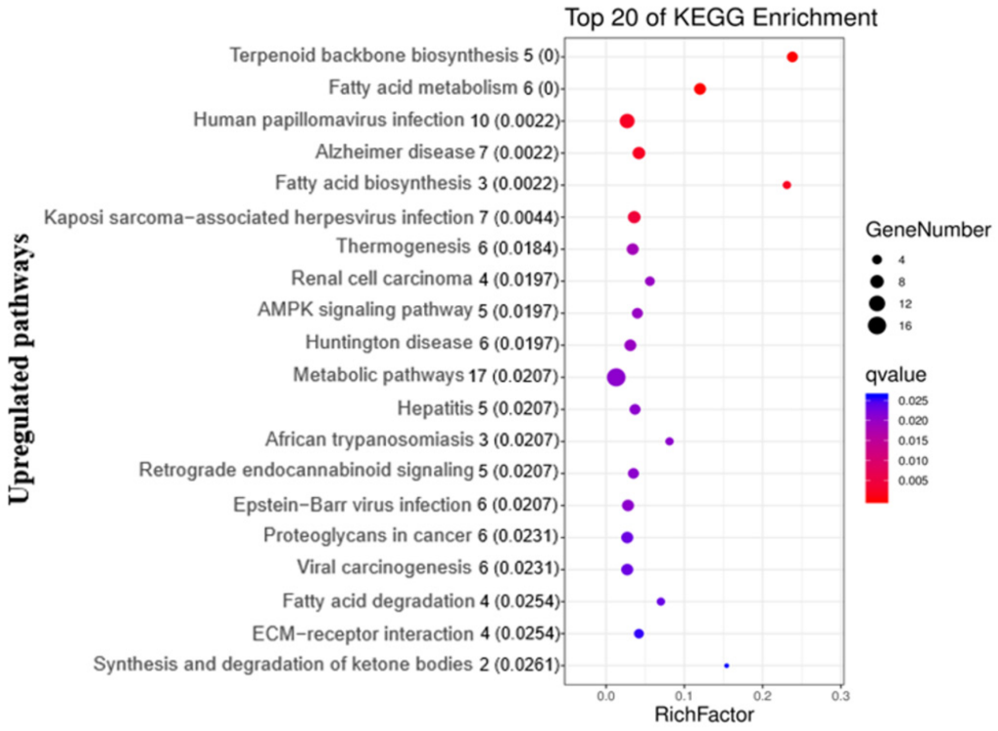

Supplementary Figure S3. KEGG of the upregulated mRNAs with the top 20 enrichment. Bubble color and size correspond to the Q value and gene number enriched in the pathway. The rich factor indicates the ratio of the number of DEGs mapped to a certain pathway to the total number of genes mapped to this pathway.

Supplementary Table S1. (**A**) Primers for qPCR, related to experimental procedures.

qPCR primers for analysis of mRNA (siCDR1as)

Gene name	Forward 5'-3'	Reverse 5'-3'
HAPLN4	GGGATGCCTCCCTGGTTCTC	TGAGCCTCGGCGAAGGTTAG
ANPEP	GCCGCAAGAACACTACTGGC	AGGGATGTTCTCCGGCCTTG
SMARCB1	AAGATACGCGACCAGGACCG	CCCTCCGAGCCAGAAGATGG
PAMR1	CCTGGACAGCACCGGATCTT	ATTTTCGCATAGCGCCCGTG
SNX27	CTTCGAGAGGGTGTTCTGCGA	ATCCCTCTGCTGTGACCTCG
ANGPT1	CAATGGGGGAGGTTGGACTGT	GGCTCGGTTTCCTTCCCAGT
E2F2	CACCCAGATGTACCCGCAGA	CACTTCCCCTTGGGAGTCCG
CCN1	CCAAGGGGCTGGAATGCAAC	TTGGGGGCACAGAGGAATGC
FGFR1	CTTTTCCGCCAACTCGCGCT	GAGGAGACACTTCCGGCTCC
MEF2C	CAGGTGGTCTGATGGGTG	GTCTGGTTTACGGTTAT
MyoD	GTGCAAACGCAAGACGACTA	GCTGGTTTGGGTTGCTAGAC
GAPDH	GCAAGTTCCACGGCACAG	GGTTCACGCCCATCACAA

Supplementary Table S1. (**B**) qPCR primers for analysis of miRNA (siCDR1as).

Gene name	Primers
miR-107-3p	AGCAGCATTGTACAGGGCTAT
miR-125-5p	TCCCTGAGACCCTAACTTGT
miR-27a-3p	TTCACAGTGGCTAAGTTCCG
miR-140-5p	CAGTGGTTTTACCCTATGGTAG
miR-29a-3p	TAGCACCATCTGAAATCGGTT
miR-143-3p	TGAGATGAAGCACTGTAGCTCG
miR-378-5p	TGGTAGACTATGGAACGTAGG
miR-140-3p	ACCACAGGGTAGAACCACGGAC
miR-184	TGGACGGAGAACTGATAAGGGT
miR-26a-5p	TTCAAGTAATCCAGGATAGGCT

U6	GAGGGCCTATTTCCCATGATTCC

Supplementary Table S2. 43 miRNAs that are critical for muscle cells after inhibition of CDR1as.

miRNAs	siCDR1as_readcount	siNC_readcount	Log2│FoldChange│	pval	padj
chi-miR-17-5p	3001.248	1810.237	0.72912	2.60E-08	3.12E-06
chi-miR-20a-5p	7199.622	4354.877	0.72503	2.78E-07	2.23E-05
novel_52	1128.806	613.921	0.87711	4.69E-07	2.82E-05
chi-miR-140-5p	2204.859	1439.661	0.61501	5.54E-06	0.000133
chi-miR-222-3p	9959.478	6640.695	0.58454	3.94E-06	0.000133
chi-let-7i-3p	367.0999	223.6484	0.71562	9.32E-06	0.000186
chi-miR-27a-3p	84563.05	57668.11	0.55225	8.88E-06	0.000186
chi-miR-19a	348.086	134.4014	1.3685	1.24E-05	0.000229
chi-miR-191-5p	6879.281	4573.587	0.58895	3.03E-05	0.000519
chi-miR-362-5p	764.6337	449.3501	0.76461	3.87E-05	0.000619
chi-miR-18a-5p	246.8867	144.2109	0.77112	0.000187	0.002495
chi-miR-15a-5p	359.2752	210.8023	0.76674	0.00126	0.014397
chi-miR-125a-5p	5395.456	3275.853	0.71978	0.001574	0.015266
chi-miR-125b-5p	62585.56	44942.4	0.47776	0.001648	0.015266
chi-miR-29a-3p	24037.27	18357.92	0.38886	0.001478	0.015266
novel_78	296.5034	198.6685	0.57919	0.001681	0.015266
chi-miR-374b-5p	5091.916	3688.704	0.46489	0.001843	0.015304
novel_31	1233.08	848.6147	0.54022	0.002001	0.016005
chi-miR-93-5p	8689.138	6836.243	0.3461	0.002745	0.021251
chi-miR-30e-5p	12620.17	9152.339	0.46333	0.002941	0.022061
chi-miR-199a-5p	68943.3	50479.2	0.44975	0.003504	0.025483
chi-miR-365-3p	1554.539	994.1919	0.64332	0.003964	0.027983
chi-miR-181b-5p	639.2447	480.2985	0.41174	0.004109	0.028179
chi-miR-874-3p	65.97939	35.72098	0.8767	0.005404	0.033985
chi-miR-146a	1106.627	862.9648	0.3581	0.005852	0.035113
chi-miR-30f-5p	235.0536	167.7368	0.49004	0.007716	0.044094
chi-miR-107-3p	1138.959	855.4164	0.4132	0.008342	0.04656
chi-miR-151-3p	19688.77	37772.71	-0.93987	3.45E-10	8.29E-08
chi-miR-140-3p	11045.99	15935.61	-0.52868	2.44E-06	9.75E-05
chi-miR-379-5p	2243.846	3453.597	-0.6214	2.20E-06	9.75E-05
chi-miR-143-3p	1027419	1649770	-0.68324	5.35E-06	0.000133
chi-miR-411a-5p	2529.26	4076.524	-0.68856	5.14E-06	0.000133
chi-miR-148a-3p	224034.2	369598	-0.72223	9.33E-05	0.0014
chi-miR-493-3p	273.7732	430.8949	-0.64994	0.000111	0.00156
novel_48	685.3696	1041.922	-0.60186	0.000232	0.002927
chi-miR-1388-5p	397.6739	564.416	-0.50206	0.000924	0.011083

chi-miR-218	563.6718	863.5813	-0.6145	0.001718	0.015266
chi-miR-26a-5p	434.6935	594.9322	-0.4532	0.001509	0.015266
chi-miR-99b-3p	251.3425	374.3879	-0.56933	0.001849	0.015304
chi-miR-184	127.8901	194.93	-0.60921	0.004527	0.029837
chi-miR-708-3p	446.1509	612.6852	-0.45456	0.0046	0.029837
chi-miR-24-3p	59250.53	76802.59	-0.37432	0.005523	0.033985
chi-miR-106b-3p	1026.171	1415.912	-0.46227	0.006238	0.036516

Supplementary Table S3. The deregulated expression of mRNAs in microarray in SMSCs (siCDR1as).

Gene symbol	Gene ID	Regulation	log2 Fold change	p value
HAPLN4	ENSCHIG00000017398	Up	0.319595	0.000607
PAMR1	ENSCHIG00000018319	Up	0.240432	0.026947
ANPEP	ENSCHIG00000010000	Up	0.220042	0.003457
SNX27	ENSCHIG00000021069	Up	0.179217	0.040782
SMARCB1	ENSCHIG00000019816	Up	0.349109	0.003646
ANGPT1	ENSCHIG00000015819	Down	-0.223933857	0.046862
E2F2	ENSCHIG00000007326	Down	-0.38529	0.016679
CCN1	ENSCHIG00000027269	Down	-0.20456	0.048326
FGFR1	ENSCHIG00000011006	Down	-0.133506628	0.046732577
MEF2C	ENSCHIG00000016206	Down	-0.211567565	0.035057
